# Advances in Research on the B-Lineage Transcription Factor EBF1 in Solid Tumors

**DOI:** 10.3390/ijms26115203

**Published:** 2025-05-28

**Authors:** Qinghua Li, Yanchuan Zhang, Guojing Xie, Junhao Cui, Ping Leng

**Affiliations:** Chongqing Key Laboratory of Sichuan-Chongqing Co-Construction for Diagnosis and Treatment of Infectious Diseases Integrated Traditional Chinese and Western Medicine, Sichuan Key Laboratory of Medical Molecular Testing, College of Medical Technology, Chengdu University of Traditional Chinese Medicine, Chengdu 611137, China; leqinghua@foxmail.com (Q.L.); zhangyanchuan1226@foxmail.com (Y.Z.); xieguojing456@163.com (G.X.); c15615615@foxmail.com (J.C.)

**Keywords:** EBF1, solid tumor, signaling pathway, oncogene, tumor suppressor gene

## Abstract

Early B-cell factor 1 (EBF1) is a crucial transcription factor that governs the development and differentiation of B lymphocytes. Furthermore, it is essential in developing multiple organs and tissues. The functional dysregulation of EBF1 expression is intricately associated with the occurrence, recurrence, and treatment resistance of B-lineage acute lymphoblastic leukemia. In recent years, EBF1 has demonstrated a more intricate and multifaceted role in solid tumors. It does not immutably adhere to the conventional classification of tumor suppressor genes. On the contrary, EBF1 is a flexible regulatory factor that exhibits diverse functional characteristics and regulatory models according to the different types of tumors and their microenvironment differences. This review elucidates the unique function of EBF1 in various solid tumors and associated signaling pathways, offering a theoretical foundation for a thorough comprehension of EBF1’s intricate roles in solid tumor development.

## 1. Introduction

In the complex biological processes of carcinogenesis, the aberrant expression of transcription factors, characterized by their overactivation or functional inactivation, frequently constitutes pivotal molecular events that propel tumor initiation, malignant advancement, and disease progression. Recent findings indicate that early B-cell factor 1 (EBF1), a transcription factor essential for cell differentiation and maturation, exhibits a comparable yet more intricate and variable mechanism of action in solid tumors compared to hematological cancers.

EBF1 is located in the region of chromosome 5q33.3 and belongs to the EBF family. The family contains four members: EBF1, EBF2, EBF3, and EBF4. They are highly conserved and have atypical zinc fingers and helix–loop–helix motifs. The EBF1 protein consists of four modular domains with different structures and functions, including an N-terminal DNA binding domain (DBD), an Ig-like/plexins/transcription factors (IPT) domain, an atypical helix–loop–helix (HLH) motif, and a C-terminal domain (CTD) [1]. The EBF1 protein interacts directly with DNA by a distinctive ‘zinc knuckle’ binding motif situated in the DBD, and the IPT domain is involved in DNA binding and/or dimerization [2,3]. The HLH motif is related to the stability of the dimer, while the less conserved COOH-terminal domain is connected to transcriptional activation [4,5]. Subsequent research has demonstrated that the CTD of EBF1 is essential for binding to specific sites on the genome and inducing DNase hypersensitive sites and is associated with methylation [6,7]. EBF1 lacking the CTD is significantly less effective than wild-type EBF1 in facilitating pre-B cell differentiation and forcing B cell fate [6,8].

The primary function of EBF1 is to maintain normal B cell development, which is related to the coordination of cell proliferation, survival, and differentiation in multiple stages of B lymphocyte production and is essential for the proliferation, survival, and signal transduction of pro-B cells and peripheral B cell subsets (including B1 cells and marginal zone B cells) [9,10]. EBF1 collaborates with other transcription factors to construct a complex regulatory network, which regulates gene expression during B cell development and restricts cell differentiation to other lineages. Knockout of EBF1 in lymphocyte progenitor cells can retain the developmental potential of T cells and myeloid cells [11,12,13]. Animal experiments have also shown that EBF1 is required for B cell differentiation [14]. EBF1 regulates B cell development in a dose-dependent manner. If its expression is defective or insufficient, it will block B cell lineage differentiation at an early stage and lead to transcriptional defects of downstream B cell differentiation-related target genes [15,16]. However, in some instances of acute lymphoblastic leukemia (ALL), aberrant expression of EBF1 is frequently detected, adversely impacting the normal development and differentiation of B cells and associated with the occurrence, drug resistance, and recurrence of ALL [17,18,19]. It can be seen that EBF1 is a crucial factor in the development of normal B cells. This transcription factor is often dysregulated in hematological malignancies, hence driving the development of leukemia.

Subsequent studies have found that EBF1 is intimately related to physiological and pathological processes such as adipocyte differentiation [20], bone metabolism [21,22], nervous system development [23], and tumorigenesis [24]. The functional inactivation of EBF1 can result in losing control of these developmental pathways, leading to tumor phenotypes. Inactivation of EBF3 has been detected in brain tumors, liver carcinoma, colon cancer, breast cancer, head and neck squamous cell carcinoma, and metastatic prostate cancer. This abnormal expression may result from gene deletion, mutation, and promoter hypermethylation [24]. EBF3 and EBF1 are members of the EBF family; therefore, it is imperative to monitor the status of EBF1 in malignancies. Still, the cancer-related research on EBF1 is predominantly focused on the hematological system. In recent years, there has been an increased examination of the association between EBF1 and solid cancers. It is involved in the initiation and progression of tumors through multiple mechanisms, and its function is closely related to the specific location of tumors. This review systematically summarizes the role of EBF1 in various solid tumors and elaborates on its associated pathways leading to malignant behavior, thereby providing a comprehensive perspective for further revealing the mechanism of EBF1 in solid tumors.

## 2. The Function of EBF1 in Solid Tumors

EBF1 plays a pivotal role in developing various tissues, particularly in lineage commitment and maturation of B lymphocytes, where its regulatory function is well established. Early studies generally suggested that EBF1 functions as a tumor suppressor in hematological malignancies. However, recent literature indicates that its role is context-dependent, and in certain specific types of hematologic cancers, EBF1 may also promote tumor progression [25,26]. In contrast, the function of EBF1 in solid tumors exhibits even greater heterogeneity. Evidence has shown that EBF1 can exert either tumor-suppressive or oncogenic effects across different solid tumors through various signaling pathways, with its molecular mechanisms displaying notable tissue specificity and contextual dependency. The related research progress is summarized in Table 1.

### 2.1. Breast Cancer

EBF1 plays a direct role in breast cancer (BC) progression by modulating tumor metabolism-related pathways. Qiu et al. reported that EBF1 is significantly overexpressed in triple-negative breast cancer (TNBC) and suppresses the co-activator function of histone acetyltransferase p300 by forming a complex with p300 and HIF1α [27]. This EBF1-p300-HIF1α complex inhibits HIF1α transcriptional activity, thereby impeding the full activation of its downstream target genes. This process helps maintain mitochondrial homeostasis in TNBC cells, inhibits cell death caused by excessive mitochondrial autophagy, and promotes TNBC cell proliferation, migration, and disease progression. Notably, the study also revealed that HIF1α can positively regulate EBF1 promoter activity, thereby upregulating EBF1 expression and establishing a positive feedback loop that reinforces the malignant phenotype of TNBC. Beyond its role in tumor cell metabolism, EBF1 also contributes to remodeling the tumor microenvironment (TME), particularly through the activation of cancer-associated fibroblasts (CAFs)—key stromal components whose increased presence is closely linked to BC progression, invasion, and metastasis. One study demonstrated that EBF1 is a critical mediator in the upregulation of FAM171A1 expression by the lncRNA SNHG14 [28]. Specifically, SNHG14 directly binds to the EBF1 protein and promotes transcription of the FAM171A1 gene by recruiting or stabilizing EBF1. This SNHG14/EBF1/FAM171A1 signaling axis has been shown to transform normal fibroblasts (NFs) into pro-tumorigenic CAFs, thereby fostering a microenvironment that supports tumor growth and metastasis.

Genetic variation in the EBF1 gene has also been implicated in BC susceptibility. A genome-wide association study (GWAS) first identified the single-nucleotide polymorphism (SNP) rs1432679, located within the EBF1 locus, as a novel susceptibility marker for TNBC [47]. It is hypothesized that this variant may influence TNBC risk by altering EBF1 expression or function. Notably, rs1432679 is also associated with mammographic density, an established, independent risk factor for BC, further supporting a potential link between EBF1 and BC susceptibility [48]. Additionally, whole-exome sequencing of clinical samples revealed somatic mutations in EBF1 in bilateral BC tissues from a single patient, identifying EBF1 as a potential cancer driver gene in synchronous bilateral BC [49]. While these findings suggest a connection between EBF1 variants and BC pathogenesis, current evidence remains insufficient to justify their use as standalone markers for individualized risk prediction. Further functional studies are needed to clarify how these genetic alterations impact EBF1 activity and its downstream oncogenic pathways.

**Figure 1:**
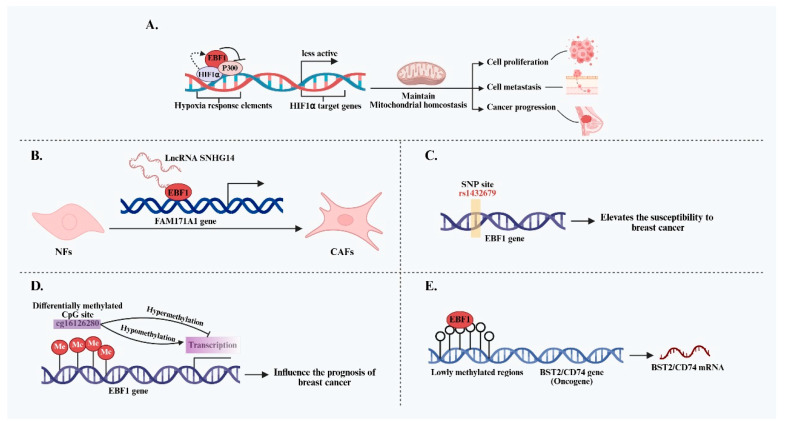
Summary of the regulatory mechanisms of EBF1 in the initiation and progression of BC. (**A**) EBF1 maintains mitochondrial homeostasis by inhibiting p300-mediated HIF1α transcriptional activity and forms a positive feedback loop with HIF1α, thereby promoting the progression of TNBC. (**B**) EBF1 promotes the transformation of NFs into CAFs by mediating the SNHG14-FAM171A1 signaling axis, thereby remodeling the BC microenvironment. (**C**) The SNP site rs1432679 within the EBF1 gene locus is closely associated with BC susceptibility. (**D**) The methylation level of the CpG site cg16126280 within the EBF1 gene is negatively correlated with its expression and is closely associated with the prognosis of BC patients. (**E**) EBF1 enriched in lowly methylated regions may affect the expression of key oncogenes by regulating local methylation status.

Epigenetic modification is a key mechanism underlying tumor phenotype development, and growing evidence suggests that EBF1 plays a significant role in the dynamic regulation of DNA methylation in BC. As early as 2013, EBF1 was shown to co-localize and directly interact with TET Methylcytosine Dioxygenase 2 (TET2) in the nucleus, collaboratively mediating DNA demethylation [50]. This interaction provides a direct mechanistic basis for EBF1-driven regulation of gene expression through modulation of DNA methylation. Genome-wide methylation analyses have identified widespread lowly methylated regions (LMRs)—rich in transcription factor binding sites—across major BC subtypes. Notably, EBF1 binding motifs in basal-like BC are significantly enriched in these LMRs [51]. Moreover, methylation levels at these EBF1 binding sites are inversely correlated with the expression of nearby genes, including oncogenes BST2 and CD74, both associated with poor prognosis. These findings strongly suggest that EBF1 may influence the expression of critical genes by binding to LMRs and modulating local DNA methylation, thereby shaping the biological behavior of specific BC subtypes. The expression of EBF1 is also subject to epigenetic regulation. A key differentially methylated CpG site within the EBF1 gene, cg16126280, has been identified, with methylation levels inversely correlated with EBF1 mRNA expression in breast tissue [52,53]. Interestingly, the prognostic significance of cg16126280 methylation appears to be age-dependent. In younger patients, hypermethylation is associated with improved survival, whereas in older patients, it correlates with poorer outcomes [52]. This age-specific pattern underscores the complexity of EBF1 epigenetic regulation and its potential differential prognostic implications across physiological contexts. In summary, EBF1 performs multifaceted functions in BC, exhibiting apparent subtype specificity. It influences disease progression and patient prognosis through diverse mechanisms, including metabolic regulation, tumor microenvironment remodeling, genetic variation, and epigenetic modification (Figure 1). A comprehensive, mechanistic understanding of EBF1 is needed to support its translational potential as a therapeutic target and prognostic biomarker in BC.

### 2.2. Gastric Cancer

**Figure 2:**
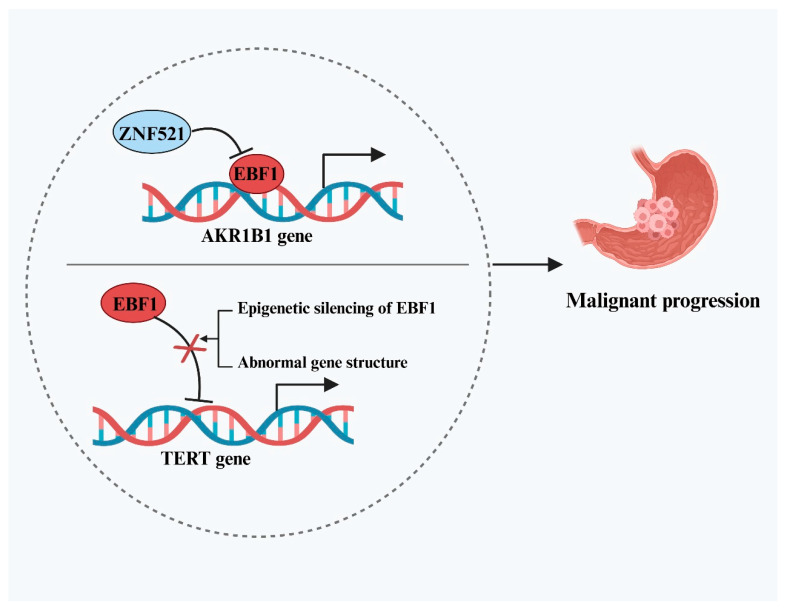
Dual tumor-suppressive mechanisms mediated by EBF1 and their dysfunction in GC. The upper half of the circle illustrates that EBF1 exerts its tumor-suppressive effect by inhibiting AKR1B1 expression, a mechanism that can be attenuated by the upregulation of ZNF521, thereby promoting GC progression. The lower half of the circle depicts that epigenetic silencing and structural abnormalities of the EBF1 gene can lead to loss of its function, disrupting the transcriptional repression of TERT and ultimately driving the progression of GC.

EBF1 exerts its anti-cancer effects in gastric cancer (GC) primarily by transcriptionally repressing key oncogenes. Cheng et al. [29] demonstrated that EBF1 directly binds to and downregulates the expression of aldo-keto reductase family 1 member B1 (AKR1B1), thereby inhibiting GC cell proliferation, migration, and invasion. However, the study also revealed that elevated levels of another transcription factor, zinc finger protein 521 (ZNF521), in tumor tissues could indirectly promote AKR1B1 expression by suppressing EBF1. This regulatory axis diminishes the tumor-suppressive activity of EBF1 and facilitates cancer progression, highlighting the complexity of upstream modulation within the EBF1-associated anti-tumor network. In addition to targeting AKR1B1, EBF1 also influences tumor progression by interfering with telomerase activity. Reactivation of the telomerase catalytic subunit (TERT) is a critical mechanism by which cancer cells bypass senescence and attain unlimited proliferative capacity, a hallmark of many malignancies. EBF1 has been identified as a direct transcriptional repressor of TERT [30]. Loss of EBF1 function or reduced expression is considered a key contributor to the aberrant upregulation of TERT in GC. This dysregulation arises through multiple mechanisms, primarily including epigenetic silencing and structural abnormalities of the gene. Epigenetic silencing involves hypermethylation of the EBF1 promoter and repressive histone modifications. Structural alterations may include mutations within the EBF1 DNA-binding domain or deletions and rearrangements near the TERT regulatory region, hindering EBF1’s ability to bind the TERT promoter. These events collectively diminish EBF1-mediated repression of TERT, resulting in enhanced telomerase activation and genomic instability, driving sustained proliferation and malignant progression of GC cells. In summary, EBF1 exerts potent tumor-suppressive effects in GC by targeting both AKR1B1 and TERT (Figure 2), and restoring EBF1 expression represents a promising strategy for targeted therapy.

### 2.3. Colorectal Cancer

**Figure 3:**
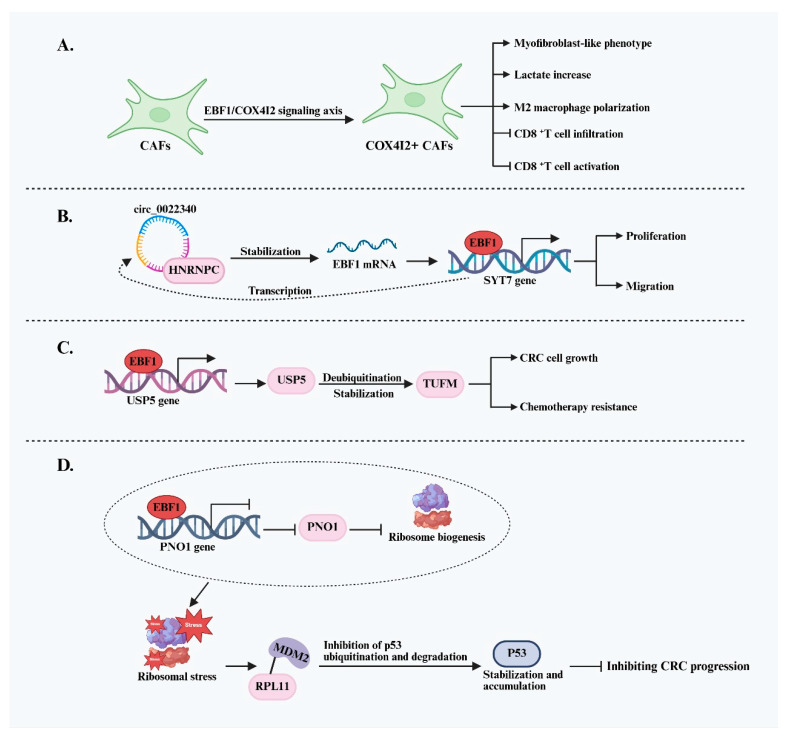
Summary of the oncogenic and tumor-suppressive mechanisms of EBF1 in CRC. (**A**) EBF1 promotes metabolic reprogramming and immune suppression by activating COX4I2+ CAFs, thereby establishing an immune-evasive microenvironment and reducing the responsiveness of CRC to immunotherapy. (**B**) circ_0022340 enhances the stability of EBF1 mRNA through HNRNPC, leading to transcriptional activation of SYT7 and driving CRC cell proliferation and migration. (**C**) EBF1 upregulates USP5 expression to stabilize TUFM protein, promoting CRC cell growth and enhancing chemoresistance. (**D**) EBF1 suppresses PNO1 expression to induce ribosomal stress, thereby activating the p53 signaling pathway and exerting a tumor-suppressive effect in CRC.

In colorectal cancer (CRC), the role of EBF1 appears to be context-dependent and functionally dualistic. Evidence suggests that EBF1 can act as a tumor suppressor and a promoter of tumor progression, depending on the cellular environment and underlying molecular networks (Figure 3). Several studies have highlighted the oncogenic potential of EBF1 in CRC, demonstrating its involvement in reshaping the TME and directly regulating the biological behavior of cancer cells. Li et al. [31] investigated the role of CAFs in CRC progression and identified a distinct CAF subpopulation (COX4I2+ CAFs) regulated by the EBF1-COX4I2 signaling axis. In this context, EBF1 was shown to bind to the promoter of COX4I2 directly, enhancing its transcription and contributing to tumor-supportive CAF activation. CAFs characterized by activation of the EBF1/COX4I2 axis exhibit a myofibroblast-like phenotype and undergo significant metabolic reprogramming, shifting from mitochondrial respiration to glycolysis, which results in elevated lactate production. This phenotypic and metabolic transformation endows COX4I2+ CAFs with strong immunosuppressive properties. These CAFs contribute to the formation of an immunosuppressive tumor microenvironment through multiple mechanisms: promoting M2 macrophage polarization, physically obstructing CD8+ T cell infiltration into the tumor core, and inducing early CD8+ T cell dysfunction via the MHC-I signaling pathway. Collectively, these effects markedly impair the responsiveness of CRC patients to immune checkpoint inhibitor (ICI) therapy, underscoring the potential role of EBF1 in mediating treatment resistance.

Beyond its role in shaping the TME, EBF1 also contributes directly to tumor progression within CRC cells. One study identified the oncogenic circular RNA circ_0022340 as a regulator that enhances EBF1 mRNA stability by recruiting heterogeneous nuclear ribonucleoprotein C (HNRNPC), indirectly increasing EBF1 protein levels. Elevated EBF1 subsequently binds to the promoter of synaptotagmin 7 (SYT7) and activates its transcription, leading to increased CRC cell proliferation and migration [32]. Additionally, EBF1 has been implicated in maintaining protein homeostasis in tumor cells, further supporting its potential role in promoting CRC progression. EBF1 has been shown to directly bind to a specific region of the ubiquitin-specific peptidase 5 (USP5) promoter, positively regulating its expression in CRC cells. USP5, a deubiquitinating enzyme, stabilizes the Tu translation elongation factor (TUFM) by removing its ubiquitination modifications. Stabilized TUFM, in turn, promotes CRC cell growth and contributes to increased resistance to chemotherapeutic agents [33]. These findings suggest that EBF1 may support tumor cell survival and drug resistance by modulating protein degradation pathways.

Contrary to its pro-tumorigenic roles, EBF1 has also been identified as a potential tumor suppressor in CRC. Its expression is often downregulated in CRC tissues, and reduced EBF1 levels are frequently associated with poor patient prognosis. The tumor-suppressive function of EBF1 is primarily attributed to its negative regulation of partner of NOB1 (PNO1), an oncogene involved in ribosome biogenesis. Under normal conditions, EBF1 inhibits PNO1 expression, leading to impaired ribosome assembly and the induction of ribosomal stress. This stress facilitates the binding of ribosomal protein L11 (RPL11) to MDM2, thereby inhibiting MDM2-mediated ubiquitination and degradation of p53. As a result, p53 is stabilized and accumulates, subsequently activating the transcription of its downstream target, p21. Activation of the p53/p21 signaling pathway effectively suppresses tumor cell proliferation and impedes CRC progression [34,35]. In summary, EBF1 exhibits dual functionality in CRC. It can promote tumor progression and treatment resistance by modulating CAF activity, cancer cell signaling, and protein homeostasis, while also acting as a tumor suppressor by inhibiting PNO1 and activating the p53/p21 pathway under specific conditions. This functional complexity underscores the importance of elucidating the precise regulatory mechanisms and context-dependent roles of EBF1 across different CRC subtypes and disease stages, particularly when considering its potential as a therapeutic target or biomarker.

### 2.4. Bladder Cancer

Bladder urothelial carcinoma (BLCA) is a highly aggressive subtype of bladder cancer (BCa) that frequently exhibits bone metastasis. Emerging evidence implicates EBF1 as a key driver of invasion and metastasis in BLCA. In a study investigating the mechanisms underlying bone metastasis, Liu et al. [54] identified EBF1 as a positive regulator of RBP7 expression through integrated bioinformatics analysis and multilevel validation. The EBF1/RBP7 regulatory axis was suggested to influence downstream pathways, including the Th2 immune response and oocyte meiosis-related signaling, thereby enhancing the malignant potential of BLCA cells and promoting bone metastasis. These findings offer a novel molecular perspective on the metastatic behavior of BLCA and propose the EBF1/RBP7 axis as a potential prognostic biomarker and therapeutic target. Beyond its role in promoting tumor cell aggressiveness, EBF1 has also garnered attention for its involvement in modulating the tumor immune microenvironment (TIME) in BCa. Bioinformatics analyses from at least two independent studies have identified EBF1 as a central regulator of immune-related gene (IRG) transcription in BLCA [55,56]. These findings suggest that EBF1 may play a pivotal role in shaping the immune landscape of BCa, either promoting immunosuppression or immune activation, by influencing immune cell infiltration and function, ultimately driving disease progression.

**Figure 4:**
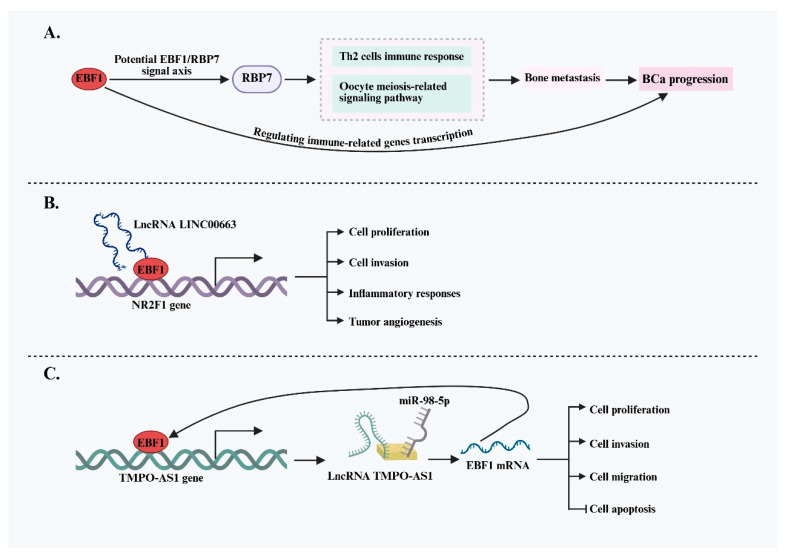
EBF1-mediated oncogenic mechanisms and regulatory network in BCa. (**A**) EBF1 may promote BCa invasiveness and bone metastasis by activating RBP7 and immune-related pathways, suggesting a potential role in malignant progression. (**B**) LINC00663 upregulates EBF1, which subsequently activates NR2F1 expression, promoting proliferation, invasion, inflammatory response, and angiogenesis in BCa cells. (**C**) EBF1 activates the oncogenic lncRNA TMPO-AS1 and forms a positive feedback loop via miR-98-5p, thereby enhancing proliferation, migration, and invasion of BCa cells while inhibiting apoptosis.

Further research has revealed a complex regulatory interplay between EBF1 and long non-coding RNAs (lncRNAs) in bladder cancer (BCa), highlighting their joint involvement in tumor pathophysiology. Zhong et al. [36] demonstrated that lncRNA LINC00663 interacts with and positively regulates EBF1 expression in BCa. This upregulation, in turn, increases the expression of the downstream target gene nuclear receptor subfamily 2 group F member 1 (NR2F1), thereby promoting cell proliferation, invasion, inflammatory responses, and tumor angiogenesis. Notably, EBF1 is not only regulated by lncRNAs but also actively regulates lncRNA expression. It has been shown that EBF1 can directly bind to the promoter region of the oncogenic lncRNA TMPO-AS1, activating its transcription [37]. The resulting high levels of TMPO-AS1 act as a competing endogenous RNA (ceRNA), sponging miR-98-5p and relieving its inhibitory effect on EBF1 mRNA. This forms a positive feedback loop, further enhancing BCa cell proliferation, migration, and invasion. These findings indicate that EBF1 plays a predominantly pro-oncogenic role in BCa and is intricately involved in the immune regulatory network (Figure 4). Elucidating the EBF1-mediated signaling pathways may deepen our understanding of BCa pathogenesis and provide valuable insights for developing novel immunotherapeutic strategies.

### 2.5. Prostate Cancer

**Figure 5:**
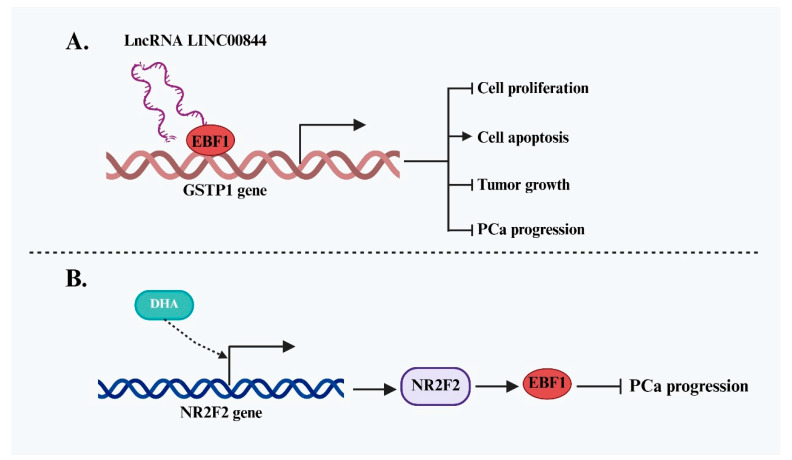
Tumor-suppressive role of EBF1 and its regulatory mechanisms in PCa. (**A**) LINC00844 recruits EBF1 to activate GSTP1 transcription, inhibiting PCa cell proliferation, promoting apoptosis, and slowing tumor growth, exerting an EBF1-dependent tumor-suppressive effect. (**B**) EBF1 is upregulated upon treatment with DHA and functions as a downstream effector of NR2F2, participating in the anti-tumor effect of DHA in PCa.

In contrast to its pro-oncogenic roles in certain malignancies, existing studies suggest that EBF1 functions predominantly as a tumor suppressor in prostate cancer (PCa) (Figure 5). A key study by Qiu et al. [38] elucidated the molecular mechanism through which EBF1 inhibits PCa progression. The authors identified lncRNA LINC00844 as a crucial mediator in this process. LINC00844 recruits EBF1 to the promoter region of glutathione S-transferase P1 (GSTP1), enhancing its transcription. GSTP1 is a well-established tumor suppressor, and its upregulation leads to reduced PCa cell proliferation and increased apoptosis, effectively impeding disease progression. Notably, the anti-cancer effect of LINC00844 was abolished upon EBF1 silencing, confirming that its tumor-suppressive function is strictly EBF1-dependent. In addition to lncRNA-mediated regulation, EBF1 expression is also modulated by other signaling pathways. Further research by Shao et al. [39] explored EBF1’s role in drug response. Treatment of PCa cells with dihydroartemisinin (DHA), an anti-tumor compound, significantly increased the expression of NR2F2, a transcription factor that directly binds to the EBF1 promoter and activates its transcription. Rescue experiments demonstrated that NR2F2 knockdown reversed the DHA-induced upregulation of EBF1, suggesting that EBF1 functions as a downstream effector of NR2F2. Although the independent anti-cancer role of EBF1 was not explicitly dissected in this study, its involvement in the NR2F2 regulatory network indirectly supports its tumor-suppressive role in PCa.

### 2.6. Others

Beyond the previously discussed cancers, the role of EBF1 in other solid tumors is gradually being uncovered, although its function exhibits considerable heterogeneity. In several malignancies, EBF1 displays apparent pro-oncogenic activity. For instance, EBF1 promotes tumor progression in osteosarcoma by regulating non-coding RNA networks. Specifically, EBF1 transcriptionally activates the lncRNA FGD5-AS1, which functions as a ceRNA by sponging miR-124-3p. This interaction relieves miR-124-3p-mediated suppression of G3BP2, leading to increased G3BP2 protein levels [40]. Upregulated G3BP2 enhances osteosarcoma cell proliferation and invasion while inhibiting apoptosis. In thyroid cancer (TC), EBF1 has been identified as a significantly upregulated oncogene. Knockdown of the tumor-suppressive lncRNA LINC00261 promotes TC cell proliferation and migration; however, this effect is reversed when EBF1 is concurrently silenced, indicating that EBF1 is a key mediator of the oncogenic pathway downstream of LINC00261 [41]. Oral squamous cell carcinoma (OSCC) is a common cancer worldwide, with recurrence and metastasis posing serious threats to patient outcomes. In OSCC, EBF1 also plays an oncogenic role, with its expression regulated by upstream lncRNAs. Specifically, the oncogenic lncRNA RP11-874J12.4, which is aberrantly overexpressed in OSCC, directly binds to and suppresses miR-19a-5p, forming a negative regulatory loop. The resulting downregulation of miR-19a-5p leads to increased expression of its target gene, EBF1. Elevated EBF1 expression subsequently promotes OSCC cell proliferation, migration, and in vivo tumor growth [42]. This study not only reveals a novel regulatory mechanism underlying OSCC tumorigenesis but also suggests a potential new target for OSCC diagnosis and therapy.

**Figure 6:**
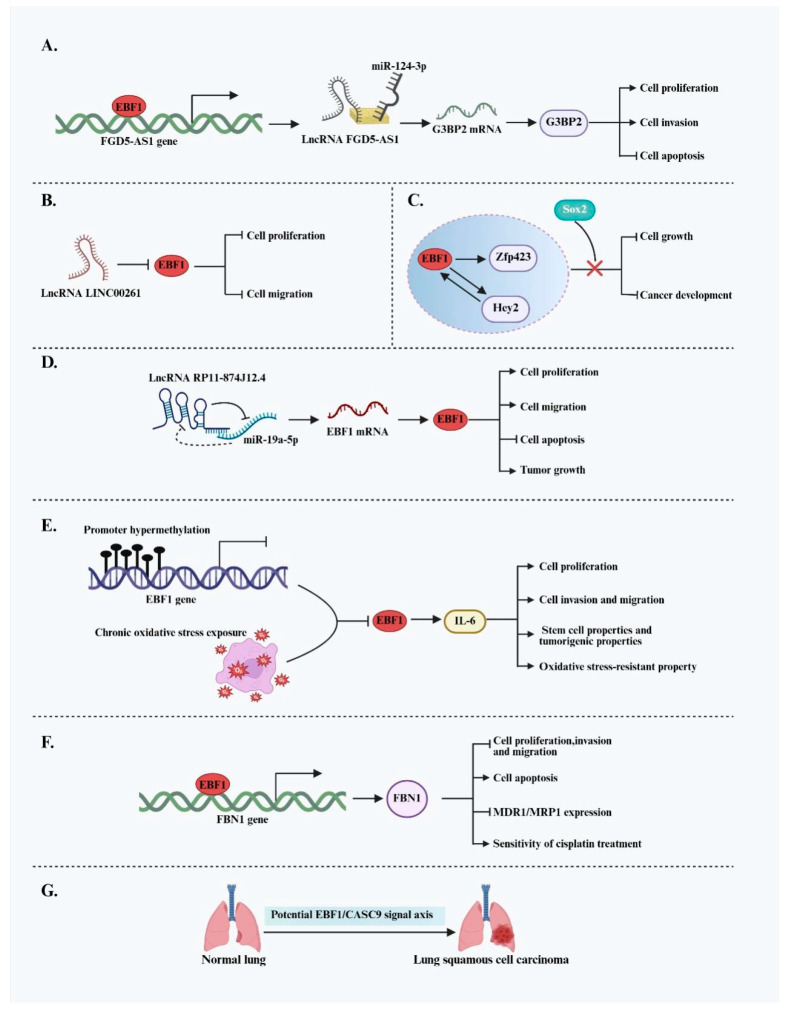
Dual functions of EBF1 and its molecular regulatory network across multiple tumor types. (**A**) EBF1 promotes the malignant progression of osteosarcoma by activating the lncRNA FGD5-AS1 and forming the FGD5-AS1/miR-124-3p/G3BP2 axis, thereby relieving the suppression of G3BP2. (**B**) The tumor-suppressive lncRNA LINC00261 inhibits EBF1 expression, attenuating EBF1-mediated proliferation and migration of TC cells. (**C**) In glioma, EBF1 suppresses cell proliferation and tumor progression by activating Hey2 and Zfp423, but this tumor-suppressive effect can be blocked by Sox2. (**D**) The oncogenic lncRNA RP11-874J12.4 upregulates EBF1 expression by inhibiting miR-19a-5p, promoting proliferation, migration, and tumor growth in OSCC. (**E**) In CCA, EBF1 expression is downregulated due to promoter hypermethylation and oxidative stress, which promotes cell proliferation, migration, invasion, stem-like properties, and tumorigenic potential and enhances the ability to adapt to oxidative stress; the tumor-promoting effects resulting from EBF1 dysregulation may be mediated through the regulation of IL-6-related signaling pathways. (**F**) EBF1 suppresses malignant behavior in CC cells by activating FBN1 expression and enhances sensitivity to the chemotherapeutic agent cisplatin. (**G**) EBF1 may be involved in the tumorigenesis of LUSC by negatively regulating the oncogenic lncRNA CASC9.

In contrast to its pro-oncogenic roles in several cancers, EBF1 exhibits clear tumor-suppressive functions in various other solid tumors. In cholangiocarcinoma (CCA), EBF1 expression is suppressed by promoter hypermethylation and chronic oxidative stress exposure. Clinical data indicate that reduced EBF1 expression is significantly associated with poor prognosis, particularly in patients with elevated oxidative stress marker 8-oxodG levels. Functionally, EBF1 downregulation promotes the acquisition of stem cell-like traits and tumorigenic potential in cholangiocytes, enhances their resistance to oxidative damage, and increases responsiveness to estrogenic stimulation—all contributing to CCA progression. Furthermore, preliminary findings suggest that EBF1 may regulate interleukin-6 (IL-6) expression, thereby modulating multiple IL-6-related downstream pathways involved in tumor progression [43,44]. In cervical cancer (CC), EBF1 acts as a transcriptional activator of fibronectin 1 (FBN1) by directly binding to its promoter. Upregulation of FBN1 suppresses tumor cell proliferation, invasion, and migration while promoting apoptosis [45]. Additionally, it downregulates the expression of multidrug resistance-associated protein 1 (MDR1/MRP1), thereby sensitizing CC cells to cisplatin treatment. These findings highlight the therapeutic potential of the EBF1/FBN1 axis in overcoming chemotherapy resistance. In glioma stem cells (GSCs), EBF1 expression is inversely correlated with Sox2. Barone et al. reported that deletion of Sox2 significantly upregulates EBF1. Activated EBF1 then promotes the expression of Hey2 and Zfp423, thereby driving GSC differentiation and inhibiting proliferation. In vivo, EBF1 overexpression markedly reduced GSC tumorigenicity, indicating its potential as a key regulator of glioma differentiation [46]. A possible tumor-suppressive role for EBF1 has also been suggested in lung squamous cell carcinoma (LUSC). Gao et al. identified a strong negative correlation between EBF1 and the oncogenic lncRNA CASC9, implying that EBF1 may modulate CASC9-mediated tumorigenesis through transcriptional repression or feedback loops [57]. However, the direct molecular interaction between EBF1 and CASC9 remains to be experimentally validated. EBF1 demonstrates highly context-dependent functions across different solid tumors, acting either as a tumor promoter or suppressor depending on the cellular context, genetic background, and tumor microenvironment (Figure 6). Therefore, for EBF1 to be considered a viable therapeutic target, it is crucial to clarify its specific roles and elucidate its upstream and downstream regulatory mechanisms in a tumor-type-specific context.

## 3. Regulation of EBF1 Expression in Solid Tumors

EBF1 expression in solid tumors is subject to multilayered regulation, encompassing epigenetic, transcriptional, post-transcriptional, and post-translational levels, reflecting a highly dynamic regulatory profile. At the epigenetic level, DNA methylation plays a key role in modulating EBF1 expression. For example, hypermethylation of the cg16126280 site in BC is associated with EBF1 downregulation and poor prognosis in an age-dependent manner [52]. At the transcriptional level, various transcription factors exert either activating or repressive effects on EBF1 expression across different tumor types. HIF1α in TNBC and NR2F2 in PCa enhance EBF1 expression [27,39], while ZNF521 in GC and Sox2 in glioma act as negative regulators [29,46], indicating that EBF1 functions as a central node at the convergence of multiple signaling pathways. Post-transcriptionally, EBF1 expression is primarily regulated by non-coding RNAs that modulate its mRNA stability and levels. For instance, circ_0022340 enhances EBF1 mRNA stability through HNRNPC [32], while TMPO-AS1 and RP11-874J12.4 relieve miRNA-mediated repression of EBF1 via ceRNA mechanisms [37,42]. Post-translational regulation also contributes to EBF1 functional modulation. SNHG14 and LINC00663 interact with EBF1 to enhance its protein stability and functional activity [28,36], whereas LINC00844 recruits EBF1 to the GSTP1 promoter to promote its transcription and exert anti-tumor effects [38]. Collectively, these multilayered regulatory mechanisms underscore the complex biological functions of EBF1 in solid tumors. Further elucidation of these processes will aid in assessing EBF1’s potential as a therapeutic target and provide a theoretical basis for tumor-type-specific interventions.

## 4. Conclusions

EBF1 is a transcription factor extensively involved in the differentiation and maturation of various cell types, with particularly critical roles in the hematopoietic system. While early studies generally identified EBF1 as a tumor suppressor in leukemia, some research has indicated that it may also exhibit oncogenic potential in certain hematologic malignancies, suggesting that its function is highly context-dependent. In comparison, the biological behavior of EBF1 in solid tumors is even more complex and heterogeneous. For instance, in CRC, EBF1 can exhibit oncogenic and tumor-suppressive effects, a duality likely driven by tumor heterogeneity. Mechanistically, EBF1 modulates tumor metabolic reprogramming, remodeling of the TME, and maintenance of protein homeostasis by regulating multiple signaling pathways. These molecular functions converge to influence key malignant phenotypes such as invasiveness, immune evasion, and treatment resistance, thereby affecting patient prognosis. Given its multifaceted role, EBF1 and its regulatory nodes hold promise as prognostic biomarkers and therapeutic targets in various solid tumors. Significantly, EBF1 loss-of-function is often associated with epigenetic silencing or genetic abnormalities in its regulatory regions. Therefore, therapeutic strategies to restore EBF1 expression or activity in specific tumor contexts may have clinical value. Furthermore, analysis based on public databases has shown that SNPs in EBF1 are closely associated with the risk of BC development, suggesting its potential clinical value in risk assessment and prognostic prediction. However, these associations require further validation in large, population-based cohorts and diverse solid tumor types to assess their generalizability. In conclusion, EBF1 is a critical regulator of solid tumor development and progression. A comprehensive understanding of its regulatory network and functional heterogeneity across tumor types will provide a robust theoretical foundation and practical framework for advancing cancer diagnosis, prognostic evaluation, and targeted therapy, ultimately benefiting a broader patient population.

## Figures and Tables

**Table 1:** The expression of EBF1 and its main function in some solid tumors.

Cancer	Expression in Solid Tumor	Tumor-Related	Regulatory Pathway	Main Biological Function	Refs.
Breast cancer	↑	pro-tumor	EBF1/p300/HIF1α	Maintain mitochondrial homeostasis to prevent cell death and promote proliferation and invasion in TNBC cells.	[27]
	↑	pro-tumor	LncRNA SNHG14/EBF1/FAM171A1	Drive the transdifferentiation of NFs into CAFs.	[28]
Gastric cancer	Not applicable	anti-tumor	ZNF521/EBF1/AKR1B1	Loss of ZNF521 upregulates EBF1 to suppress AKR1B1 and attenuate the proliferation, migration, and invasion of gastric cancer cells.	[29]
	↓	anti-tumor	EBF1/TERT	Inactivation of EBF1 leads to the reactivation of the carcinogenic factor TERT.	[30]
Colorectal cancer	↑	pro-tumor	EBF1/COX4I2	EBF1 promotes the progression of CRC by upregulating COX412 expression, inducing immunosuppression within the tumor microenvironment.	[31]
	Not applicable	pro-tumor	circ_0022340/HNRNPC/EBF1/SYT7	Overexpression of EBF1 enhances CRC migration and invasion.	[32]
	Not applicable	pro-tumor	EBF1/USP5/TUFM	Enhance the expression of USP5 and promote the growth and chemotherapy resistance of CRC.	[33]
	↓	anti-tumor	EBF1/PNO1/p53	Overexpression of EBF1 led to growth inhibition, cell cycle arrest, and increased apoptosis in CRC cells.	[34,35]
Bladder cancer	Not applicable	pro-tumor	Lnc RNA LINC00663/EBF1/NR2F1	Promote malignant biological behaviors such as inflammatory response and angiogenesis.	[36]
	↑	pro-tumor	LncRNA TMPO-AS1/miR-98-5p/EBF1	Promote proliferation, migration, and invasion.	[37]
Prostate cancer	Not applicable	anti-tumor	Lnc RNA LINC00844/EBF1/GSTP1	EBF1 recruited by LINC00844 inhibits prostate cancer progression by binding and activating GSTP1 gene expression.	[38]
	Not applicable	anti-tumor	NR2F2/EBF1	Inhibit epithelial/mesenchymal transition, reduce inflammation, and promote apoptosis.	[39]
Osteosarcoma	↑	pro-tumor	EBF1/FGD5-AS1/miR-124-3p/ G3BP2	EBF1 promotes the proliferation and invasion of osteosarcoma cells by directly binding and activating FGD5-AS1.	[40]
Thyroid cancer	↑	pro-tumor	LncRNA LINC00261/EBF1	Overexpression of EBF1 reversed the inhibitory effect of silencing LINC00261 on the proliferation and migration of TC cells.	[41]
Oral squamous cell carcinoma	↑	pro-tumor	RP11-874J12.4/miR-19a-5p/EBF1	Upregulation of EBF1 expression promotes OSCC proliferation, migration, and tumor growth in vivo.	[42]
Cholangiocarcinoma	↓	anti-tumor	IL-6 related pathways	The low expression of EBF1 enhances the tumorigenic potential and is related to the poor prognosis of patients.	[43,44]
Cervical Cancer	↓	anti-tumor	EBF1/FBN1	EBF1 inhibits cervical cancer progression and cisplatin resistance by transcriptionally activating FBN1.	[45]
Glioma	Not applicable	anti-tumor	Sox2/EBF1	Overexpression of EBF1 inhibits cell proliferation, promotes differentiation, and inhibits tumor growth.	[46]

↑: EBF1 upregulation. ↓: EBF1 downregulation. “Anti-tumor” denotes its tumor-suppressive effect, and “Pro-tumor” its oncogenic role.

## Data Availability

No new data were created or analyzed in this study. Data sharing is not applicable to this article.
